# Cerebral volumetric abnormalities in Neurofibromatosis type 1: associations with parent ratings of social and attention problems, executive dysfunction, and autistic mannerisms

**DOI:** 10.1186/s11689-015-9128-3

**Published:** 2015-10-15

**Authors:** Stephan CJ Huijbregts, Marisa Loitfelder, Serge A Rombouts, Hanna Swaab, Berit M Verbist, Enrico B Arkink, Mark A Van Buchem, Ilya M Veer

**Affiliations:** Leiden Institute for Brain and Cognition (LIBC), Leiden University, Leiden, The Netherlands; Department of Clinical Child and Adolescent Studies, Leiden University, Leiden, The Netherlands; Department of Neurology, Medical University of Graz, Graz, Austria; Department of Radiology, Leiden University Medical Center, Leiden, The Netherlands; Institute of Psychology, Leiden University, Leiden, The Netherlands; Radboud University Medical Center, Nijmegen, The Netherlands; Department of Psychiatry and Psychotherapy, Division of Mind and Brain Research, Charité Universitätsmedizin Berlin, Berlin, Germany; Department of Clinical Child and Adolescent Studies–Neurodevelopmental Disorders, Faculty of Social Sciences, Leiden University, P.O. Box 9555, 2300 RB Leiden, The Netherlands

**Keywords:** Neurofibromatosis type 1, Executive and social functioning, Magnetic resonance imaging, Voxel-based morphometry, Subcortical volume, Gray matter

## Abstract

**Background:**

Neurofibromatosis type 1 (NF1) is a single-gene neurodevelopmental disorder, in which social and cognitive problems are highly prevalent. Several commonly observed central nervous system (CNS) abnormalities in NF1 might underlie these social and cognitive problems. Cerebral volumetric abnormalities are among the most consistently observed CNS abnormalities in NF1. This study investigated whether differences were present between NF1 patients and healthy controls (HC) in volumetric measures of cortical and subcortical brain regions and whether differential associations existed for NF1 patients and HC between the volumetric measures and parent ratings of social skills, attention problems, social problems, autistic mannerisms, and executive dysfunction.

**Methods:**

Fifteen NF1 patients (mean age 12.9 years, SD 2.6) and 18 healthy controls (HC, mean age 13.8 years, SD 3.6) underwent 3 T MRI scanning. Segmentation of cortical gray and white matter, as well as volumetry of subcortical nuclei, was carried out. Voxel-based morphometry was performed to assess cortical gray matter density. Correlations were calculated, for NF1-patients and HC separately, between MRI parameters and scores on selected dimensions of the following behavior rating scales: the Social Skills Rating System, the Child Behavior Checklist, the Social Responsiveness Scale, the Behavior Rating Inventory of Executive Functioning, and the Dysexecutive Questionnaire.

**Results:**

After correction for age, sex, and intracranial volume, larger volumes of all subcortical regions were found in NF1 patients compared to controls. Patients further showed decreased gray matter density in midline regions of the frontal and parietal lobes and larger total white matter volume. Significantly more social and attention problems, more autistic mannerisms, and poorer executive functioning were reported for NF1 patients compared to HC. In NF1 patients, larger left putamen volume and larger total white matter volume were associated with more social problems and poorer executive functioning, larger right amygdala volume with poorer executive functioning and autistic mannerisms, and smaller precentral gyrus gray matter density was associated with more social problems. In controls, only significant negative correlations were observed: larger volumes (and greater gray matter density) were associated with better outcomes.

**Conclusions:**

Widespread volumetric differences between patients and controls were found in cortical and subcortical brain regions. In NF1 patients but not HC, larger volumes were associated with poorer behavior ratings.

## Background

Neurofibromatosis type 1 (NF1) is a single-gene disorder affecting approximately 1 in 3500 individuals [1]. It is inherited in autosomal dominant fashion, but in about half of the affected individuals, it arises as a spontaneous mutation. The NF1 gene is located on chromosome 17 (17q11.2); it encodes for the protein neurofibromin, which is thought to act as a tumor suppressor. Neurofibromin is involved in Ras GTPase activation [2]. Ras GTPase downregulates Ras, a family of proteins involved in cell proliferation and differentiation. Thus, lack of neurofibromin due to NF1 gene defects may lead to a lack of inhibitory control over Ras, resulting in increased cell formation, migration, and differentiation. Clinical features of NF1 include café-au-lait spots, skin fold freckling, Lisch nodules, neurofibromas (i.e., Schwann cell tumors), optic pathway gliomas, and bone lesions (e.g., short stature or scoliosis) [1, 2].

Several brain abnormalities have been observed in NF1, including neoplasms, T2 hyperintensities (T2H), macrocephaly, and abnormalities in white matter (WM) integrity [3]. Macrocephaly in NF1 has mostly been attributed to increased WM [4–6]. Volumetric studies of subcortical regions are sparse: one recent study showed significantly larger thalamic and right caudate volumes in NF1 compared to healthy controls [7]. Links between neurobiological abnormalities associated with NF1 and structural brain abnormalities have not yet firmly been established, although animal studies increasingly show the importance of neurofibromin and Ras-signaling for appropriate brain development. For example, in NF1^+/−^ mice, increased interneuronal Ras-signaling causes an increase in GABA release [8]. The level of GABAergic inhibition does not only play an important role in ongoing function of neuronal networks but also affects neuronal development, for example, by modulating the length of developmental sensitive periods. In NF1, increased GABAergic inhibition may cause early closure of sensitive periods leading to altered patterning in cortical areas [2, 8].

Cognitive and social problems have also extensively been reported for NF1 patients. The most prominent findings include problems with executive functioning (e.g., working memory, inhibitory control) [9, 10] and a particularly high incidence of autistic traits (with a prevalence of up to 30 % in the severe, clinical range, and a further 25–30 % in the mild to moderate range) and ADHD (also a prevalence of up to 50 %, although it is not yet clear which proportion of those has attention deficit disorder without hyperactivity) [11–14]. Increased GABA release may not only be associated with the (social-)cognitive phenotype of NF1 through its modulatory role in activity flow in the striatum, but, as noted, may also have led to anatomical brain abnormalities, which in turn underlie the cognitive and social problems observed in NF1 [2].

Several studies tried to link the anatomical abnormalities to aspects of cognition in NF1. Although there are some exceptions (particularly concerning thalamic T2H), generally, no relationship was found between the number of T2H and cognitive deficits [3, 15].

Whereas macrocephaly itself was generally not related to cognitive outcomes either [4], there have been reports of an absence of the normal positive correlation between gray matter (GM) volume and intelligence in NF1 patients [6], of smaller superior temporal gyrus GM volume being associated with poorer performance in a social cognition task [16], and of negative correlations between callosal WM volume and cognitive outcomes [3]. Associations between GM and WM volumes and social functioning have not yet been investigated in NF1.

The present study aimed to investigate cortical and subcortical volumetric brain abnormalities in NF1 in relation to social and attention problems, social skills, autistic mannerisms, and executive functioning.

Based on the knowledge that lack of neurofibromin may lead to a lack of inhibitory control over Ras, resulting in increased cell formation, migration, and differentiation, and based on the existing evidence on volumetric brain abnormalities in NF1, it was hypothesized that subcortical volumes and whole brain gray and white matter volumes would be larger in NF1 compared to healthy controls. Based on the evidence for suboptimal cortical organization [2] and previous findings in NF1 [16], it was expected that cortical GM density would be decreased in NF1 compared to healthy controls. Furthermore, it was expected that significant group differences would exist regarding (parental reports of) executive dysfunction, social skills, social and attention problems, and autistic mannerisms. Finally, it was hypothesized that the extent of volumetric abnormalities (increases in subcortical and total WM and GM volumes, decreases in GM density) in NF1 patients would be related to the severity of parent-rated social and cognitive impairments.

## Methods

### Participants

The sample consisted of 15 NF1 patients (mean age 12.9 years, SD 2.6; median 13.1 years, range 9.3 years, 9 boys, 6 girls) and 18 healthy controls (HC, mean age 13.8 years, SD 3.6, median 12.4 years, range 9.9 years, 8 boys, 10 girls), all of whom underwent MRI scanning. Healthy controls were friends/acquaintances of the NF1 patients. All 15 NF1 patients and 12 of the HC were compared on cognitive, behavioral, and social outcomes. All NF1 patients fulfilled the diagnostic criteria specified by the National Institutes of Health Consensus Conference [1]. Of the NF1 patients, six had an official diagnosis of ADHD and two of those also had an official diagnosis of ASS. Five of these six patients used a form of methylphenidate. None of the patients had epilepsy.

### Instruments/measures

#### Behavior rating scales

Cognitive and social functioning were assessed using parental reports on several behavior rating scales. Social skills were represented by the total score (i.e., the sum of four subscales: self-control, assertion, cooperation, and responsibility) on the parent version of the Social Skills Rating System (SSRS) [17]. Social and attention problems were assessed with the Child Behavior Checklist (CBCL) [18]. Autistic mannerisms were assessed using the Social Responsiveness Scale (SRS) [19]. In contrast to all other questionnaires, higher scores in the SSRS represent better outcomes. Therefore, we reversed its scores for comparability in statistical analyses.

Executive functioning was assessed using the parent-rated Behavior Rating Inventory of Executive Function (BRIEF) [20], which contains questions about nine interrelated subdomains of EF in daily life situations, and from which the total score (the Global Executive Composite, GEC) was calculated and used in the present study. In addition, the Dysexecutive Questionnaire (DEX) was used to measure EF (total score only). The DEX is part of the Behavioral Assessment of the Dysexecutive Syndrome (BADS) [21].

#### MRI data acquisition

All subjects underwent scanning at the Leiden University Medical Center. Imaging was performed on a Philips 3 Tesla Achieva MRI scanner using an eight-channel SENSE receiver head coil (Philips Medical Systems, Best, The Netherlands). In each subject, a T1-weighted anatomical scan was acquired with the following scan parameters: 3D T1 TFE sequence, 140 axial slices, TR 9.8 ms, TE 4.6 ms, flip angle 8°, in-plane voxel size 1.16 × 0.92 mm, 1.2 mm slice thickness, no slice gap. In addition, a T2-weighted anatomical scan was acquired (52 slices, TR = shortest, TE 80 ms, flip angle 90°, in-plane voxel size: 0.43 × 0.478 mm, 3 mm slice thickness, no slice gap). All anatomical scans were reviewed by a neuroradiologist.

### MRI data analyses

#### Subcortical segmentation

FMRIB Software Library (FSL)’s Integrated Registration and Segmentation Tool (FIRST) was used to obtain volumes of subcortical gray matter regions [22]. T1 input data was normalized to the 1 mm Montreal Neurological Institute (MNI) 152 standard space using an affine transformation. Next, a subcortical mask was applied to exclude voxels outside the subcortical regions, followed by automated segmentation based on shape models and voxel intensities. Next, a boundary correction was applied to ameliorate partial volume effects, after which absolute volumes of subcortical structures were calculated, taking into account the transformations made at the first stage.

#### Voxel-based morphometry

T1 data was analyzed with FSL-VBM, a voxel-based morphometry style analysis [23], part of FSL. The following steps were performed: brain extraction [24], tissue-type segmentation, and nonlinear normalization to MNI152 standard space. The resulting images were averaged to create a study-specific template, to which the native gray matter images were then nonlinearly re-registered. These were then modulated (to correct for local expansion or contraction) by dividing by the Jacobian of the warp field. Next, the modulated segmented images were smoothed with an isotropic Gaussian kernel (sigma of 3 mm). For group comparisons, a general linear model was applied using permutation-based nonparametric testing (randomize), correcting for multiple comparisons across space (TFCE [25]), and familywise error rate (FWE) corrected *p* = .05). Age and sex were used as covariates.

#### Whole brain gray and white matter

Tissue type segmentation was carried out with FAST on the T1 scans in native space [26], and total gray and white matter volumes were calculated. Using SIENAX, the T1 scans were linearly registered to MNI152 space, yielding a global scaling value for each participant that serves as a proxy for total intracranial volume.

#### T2H

The T2 scans from NF1 patients were visually checked by a neuroradiologist for the presence of T2H. Next, hand-labeled masks were created of all voxels showing T2H.

### Statistical analyses

SPSS 21 for Windows (SPSS, Chicago, IL) was used for statistical analyses of all metrics except for the VBM analysis. The Kolmogorov-Smirnov test was used to test for distribution normality. To test for group differences in social skills, social and attention problems, autistic mannerisms, and executive dysfunction multivariate analysis of covariance (MANCOVA, correcting for age and sex) was used. To test for volumetric group differences, ANCOVA (correcting for age, sex and scaling) was chosen. One-tailed Pearson correlations were used to correlate questionnaire scores with volumetric parameters. For this, 1000 equally sized random samples were generated from the original sample (bootstrap method) to robustly estimate the standard error. Confidence intervals of the correlation coefficients were used for decision of significance.

### Ethics statement

The study was conducted in accordance with the Declaration of Helsinki, and all procedures were reviewed and approved by the Ethics Committee of Leiden University, Faculty of Social Sciences, Department of Education and Child Studies, the Department of Radiology at Leiden University Medical Center, and the Medical Ethics Committee at Leiden University Medical Center (CCMO NL30665.058.09/P09.221/SH/sh). Written informed consent was obtained from all participants and their parents/guardians.

## Results

### Behavioral, cognitive, and social outcomes

Although total group samples did not differ significantly with respect to age or gender distribution, there were differences between NF1 patients and those HC with scores available on the behavior rating scales (mean age HC 15.3 years, SD 3.4; *t* = 2.101, *p* = .046; 2 boys, 10 girls, *χ*^*2*^(1) = 5.185, *p* = .023). Age and gender were introduced as covariates in statistical analyses. As shown in Table 1, NF1 patients and controls differed significantly on all but one of the cognitive and social scores (multivariate effect for group: *F*(6, 16) = 3.086, *p* = .033; all univariate differences to the disadvantage of NF1 patients).

**Table 1:** Mean scores (SD) on social, cognitive, and behavioral outcome measures (NF1 = 15; HC = 12), corrected for age (MANOVA using group and sex as factors and age as covariate)

	NF1	HC	F	*p*	η_*p*_^2^
SSRS total^a^	44.7 (13.7)	67.6 (7.6)	10.786	0.004	0.339
CBCL social problems	8.6 (4.7)	1.5 (1.5)	12.095	0.002	0.365
CBCL attention problems	9.5 (4.3)	2.4 (3.7)	5.387	0.030	0.204
SRS autistic mannerisms	24.9 (6.0)	13.6 (1.9)	11.486	0.003	0.354
BRIEF-GEC	149.5 (33.7)	97.4 (20.1)	8.582	0.008	0.290
DEX total	32.5 (16.8)	12.7 (14.4)	2.451	0.132	0.105

*NF1* neurofibromatosis type 1, *HC* healthy control, *SSRS* social skills rating system, *CBCL* child behavior checklist, *SRS* social responsiveness scale, *BRIEF-GEC* behavior rating inventory of executive function-global executive composite, *DEX* dysexecutive questionnaire

^a^Higher scores indicate fewer problems

### Subcortical volumes

Table 2 shows subcortical volumes for NF1 and HC. Larger volumes were found for all subcortical structures for NF1 compared to HC (FDR-corrected for multiple comparisons, *q* = .05). When adding a covariate for global scaling (serving as a proxy for total intracranial volume), group differences were still observed for the bilateral thalamus, right hippocampus, bilateral globus pallidus, and right nucleus accumbens (FDR-corrected for multiple comparisons, *q* = .05).

**Table 2:** Cortical, subcortical, and white matter volumes of NF1 patients and healthy controls (HC)

	Group	Mean	SD	*p* ^a^	*p* ^b^
Gray matter volume (unnormalised)	HC	0.743621	0.091577	.180	.348
	NF1	0.796610	0.077320		
White matter volume (unnormalised)	HC	0.582686	0.092045	.021	.041
	NF1	0.658080	0.082992		
Thalamus left	HC	8168.40	814.18	<.001*	<.001*
	NF1	9584.09	737.11		
Thalamus right	HC	7971.48	824.47	<.001*	<.001*
	NF1	9531.54	812.25		
Putamen left	HC	5306.29	687.22	.003*	.036
	NF1	6108.28	734.30		
Putamen right	HC	5342.43	724.24	.005*	.058
	NF1	6170.75	754.84		
Pallidum left	HC	1855.57	200.37	<.001*	.001*
	NF1	2225.89	274.55		
Pallidum right	HC	1862.97	212.24	<.001*	.002*
	NF1	2251.49	301.51		
Hippocampus left	HC	3845.07	568.18	.002*	.029
	NF1	4521.35	559.96		
Hippocampus right	HC	3926.33	632.01	<.001*	<.001*
	NF1	4907.53	446.63		
Caudate left	HC	3976.20	549.63	.044*	.341
	NF1	4465.80	674.85		
Caudate right	HC	4040.20	585.68	.005*	.062
	NF1	4780.13	722.43		
Amygdala left	HC	1102.04	195.87	.008*	.045
	NF1	1310.69	325.96		
Amygdala right	HC	1010.01	233.10	.030*	.135
	NF1	1216.24	393.13		
Accumbens left	HC	491.99	115.71	.018*	.068
	NF1	610.17	135.48		
Accumbens right	HC	326.92	79.81	.010*	.008*
	NF1	435.06	142.33		

*Subcortical volume significance after FDR correction (*q* <.05)

^a^Including group and sex as factors and age as covariate

^b^Including group and sex as factors and age and scaling as covariate

### Voxelwise gray matter volume differences

**Fig. 1:**
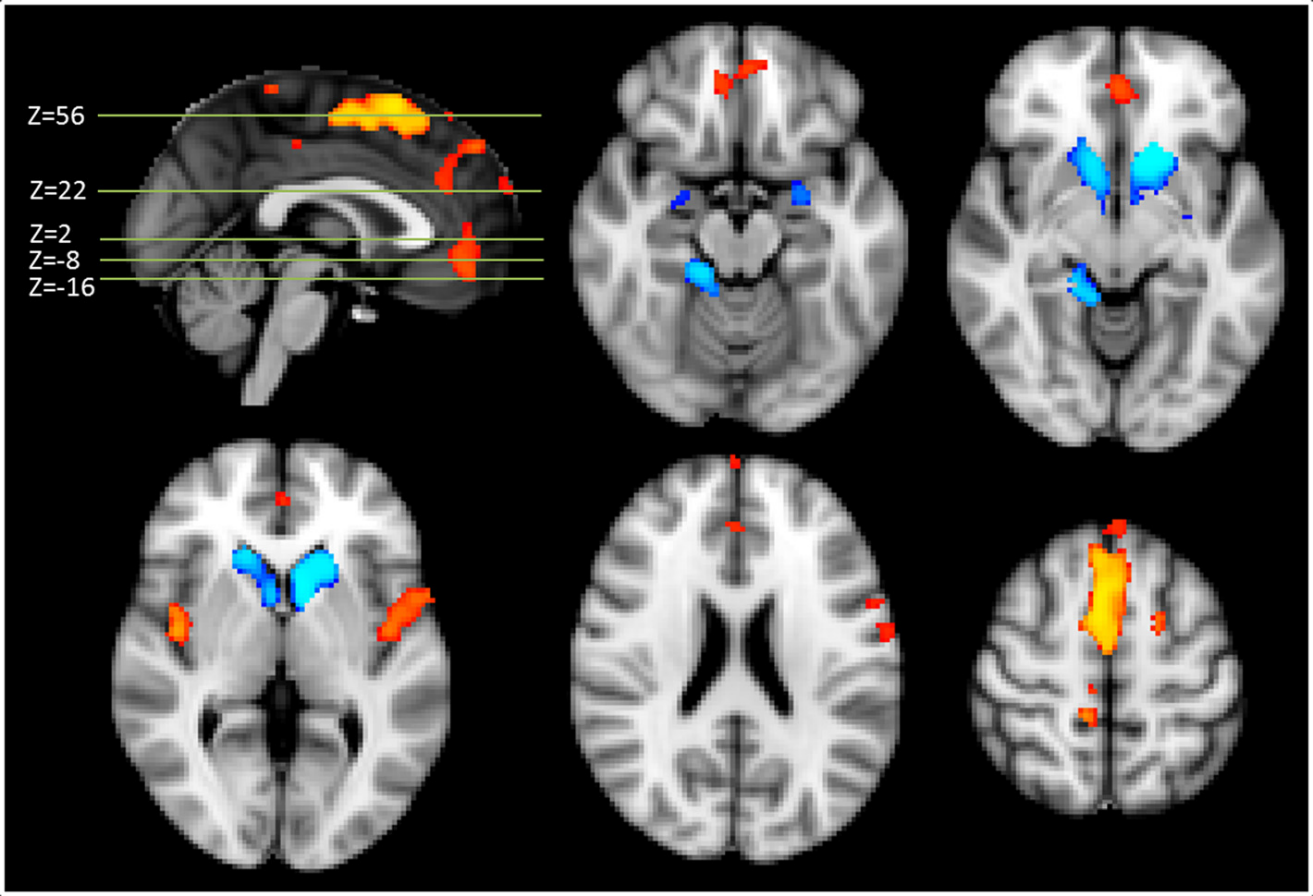
Voxel-based morphometry: differences in gray matter density between NF1 patients and healthy controls (*yellow* controls > NF1, *blue* NF1 > controls) corrected for age and sex (TFCE, FWE corrected *p* = .05). *Brighter color* indicates higher *t* scores

Figure 1 shows significant group differences regarding cortical and subcortical brain regions. GM density was found to be decreased in NF1 patients compared to controls in the bilateral pre- and right postcentral gyrus, right posterior cingulate cortex, supplementary motor area, bilateral paracingulate cortex, bilateral central opercular cortex, bilateral insular cortex, and right temporal pole. In contrast (and consistent with the FIRST-analysis), NF1 had larger GM density than HC in the bilateral caudate nucleus, bilateral putamen, right parahippocampal gyrus, bilateral amygdala, and bilateral nucleus accumbens. No differences were found considering gender. Also, there were no significant group by gender interactions.

### Total white and gray matter volume

Larger white matter (*p* = .021) but not gray matter (*p* = .18) volumes (uncorrected for total intracranial volume) were found for NF1 compared to HC (Table 2).

### T2 hyperintensities

T2H were identified in 66.7 % (*n* = 10 of 15) of the patients, of whom 33.3 % (*n* = 5) showed T2H in the thalamus, 40.0 % (*n* = 6) in the cerebellum, 26.7 % (*n* = 4) in the globus pallidus, 20.0 % (*n* = 3) in the brainstem and cortical gray matter, 6.7 % (*n* = 1) in the putamen and amygdala, and 33.3 % (*n* = 5) in the cerebral white matter. Nonparametric comparison of patients with and without T2H revealed no differences in executive dysfunction (GEC, *p* = .240; DEX, *p* = .371), attention problems (CBCL-AP, *p* = .679), social problems (CBCL-SP, *p* = .165), social skills (SSRS total, *p* = .254), or autistic mannerisms (SRS-AM, *p* = .055).

### Correlation analyses

In NF1 patients, the following subcortical volumes showed significant positive correlations with cognitive and social behavior ratings: right amygdala with EF measure BRIEF and SRS autistic mannerisms and left putamen with EF measure DEX and CBCL social problems. The only significant negative correlation for a subcortical structure was observed between the right nucleus accumbens and CBCL social problems. Local gray matter density of the precentral gyrus also showed a negative correlation with CBCL social problems, whereas white matter showed a significant positive correlation with EF-DEX and CBCL social problems.

In controls, only significant negative correlations were observed: bilateral thalamus and left nucleus accumbens were negatively correlated with CBCL-social problems, the left putamen with EF-DEX and SRS-autistic mannerisms, the bilateral hippocampus with the SSRS-total score, the left hippocampus with EF-DEX, the right caudate nucleus and left amygdala with EF-DEX, SRS-autistic mannerisms, and SSRS-total score, and the right accumbens with EF-BRIEF.

**Fig. 2:**
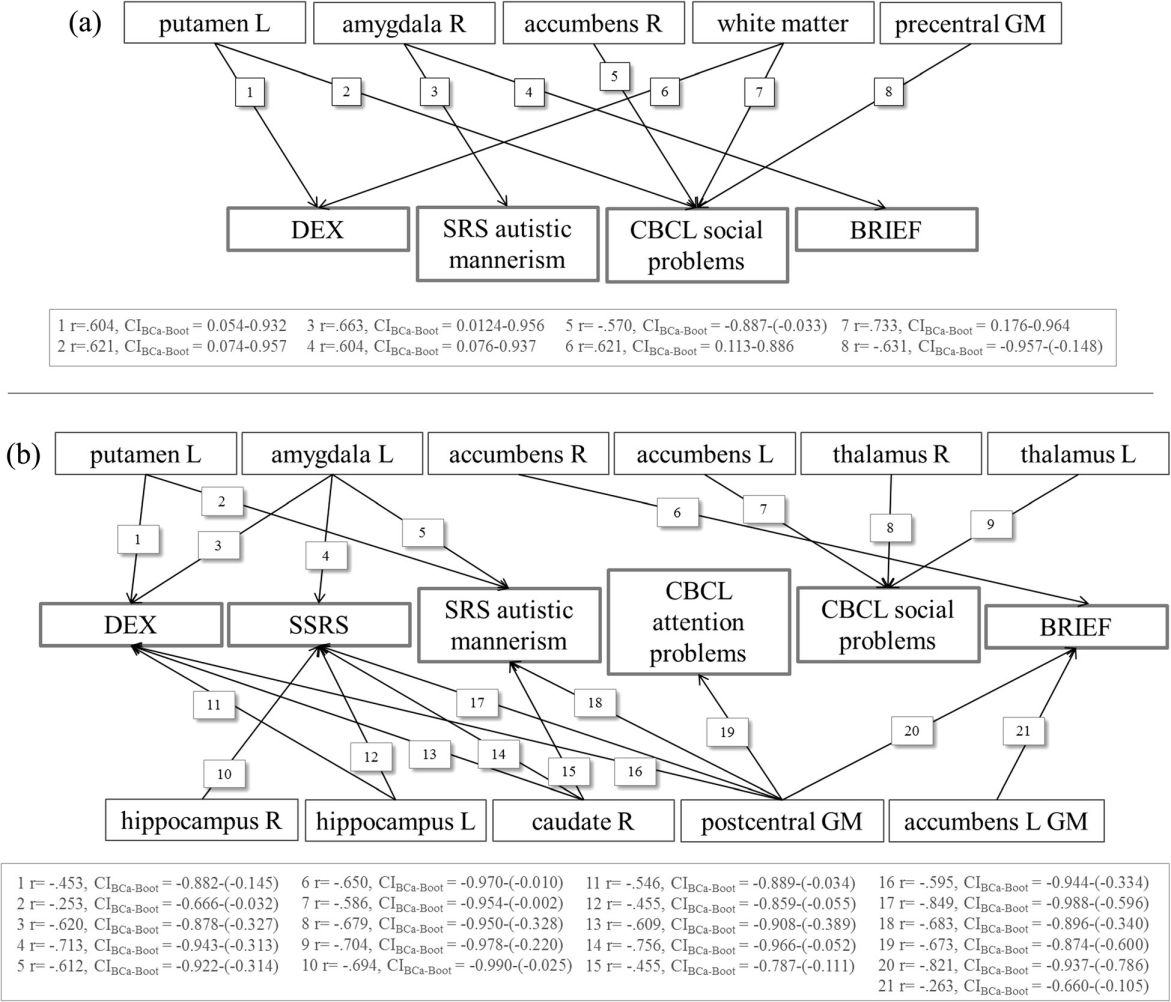
Illustration of correlations between brain volumes and executive and social outcomes in NF1 patients (**a**) and healthy controls (**b**). Listed below is the correlation coefficient, *r*, with the corresponding confidence interval, *CI*

Furthermore, local gray matter density of the postcentral gyrus showed significant negative correlations with EF-DEX and EF-BRIEF, SRS-autistic mannerisms, the SSRS-total score and CBCL-attention problems, and local gray matter density of the left nucleus accumbens showed a negative correlation with EF-BRIEF. A summary of correlations can be seen in Fig. 2.

## Discussion

This study provides indications for a neuroanatomical basis of the executive and social problems in NF1. All subcortical structures investigated (thalamus, hippocampus, amygdala, caudate nucleus, putamen, globus pallidus, and nucleus accumbens) were larger in NF1 compared to HC. NF1 patients also had larger overall WM volume compared to HC. In contrast, smaller GM densities in NF1 compared to HC were found in midline regions of the frontal and parietal lobes. NF1 patients were rated to have more social, attention, and EF problems than HC, more autistic mannerisms, and poorer social skills. Whereas among HC, larger GM volumes of cortical and subcortical structures were generally associated with better cognitive and social outcomes; the same effect for NF1 patients was only found for the precentral gyrus and the right accumbens.

These results add important information to the converging evidence on associations between volumetric properties of specific brain regions and social and cognitive functioning in healthy and clinical populations. In healthy people, larger cortical GM volumes have been associated with better cognitive outcomes rather consistently [27] and larger subcortical volumes with better social and social-cognitive outcomes [28]. In contrast, in children and adolescents with autism spectrum disorders, relative enlargements of subcortical structures (e.g., the amygdala) have been observed [29]. Though in itself relatively rare, NF1 shares a common pathophysiology, involving the Ras/mitogen-activated protein kinase (MAPK) pathway, with several other genetic syndromes. Examples include tuberous sclerosis complex, fragile X, Noonan, Costello, and Legius syndromes. Similarities between these disorders have also been observed regarding clinical features and behavioral phenotypes [30]. In fact, it has even been suggested that the syndromes characterized by dysregulation of the Ras/MAPK signaling pathway collectively account for up to 20 % of autism spectrum disorders [31].

It appears that a certain optimum regarding cellular growth is required for adequate development. In disorders such as NF1, this optimum is exceeded, resulting in less-effective connections within and between brain regions associated with cognitive and social functioning. This subsequently might lead to autism-type behavioral phenotypes.

Our findings regarding cognitive and social outcomes, as well as regarding autistic mannerisms are in accordance with previous reports [9–13]. Volumetric abnormalities (mainly enlargements) have also been reported before in NF1, both regarding whole-brain WM [4–6], and, more recently, for subcortical structures such as the thalamus and caudate nucleus [7]. In the present study, the volumetric abnormalities appear to be more widespread than previously reported and direct associations have been observed between whole-brain WM, precentral GM, and subcortical GM on the one hand and cognitive and social problems on the other in NF1.

There are only a few studies available with a somewhat comparable design, yet still, there are important differences. For example, Pride and colleagues [16] did investigate whether volumetric abnormalities were related to performance on a social cognition task in NF1 patients. Contrary to our results, no volume differences were reported for structures such as the putamen and amygdala, and subcortical volumes were not related to performance of the social cognition task in NF1. The authors did find that, in NF1, smaller superior temporal gyrus GM volume was associated with poorer task performance. However, there were important differences with respect to the outcome measures that were used (a relatively complex social-cognitive task, involving the recognition of emotions and sarcasm, versus daily life social and executive (dys-)function as measured by a series of behavior rating scales in our study) and sample characteristics. For example, mean age of their NF1 patients was 34.4 years, whereas in our study, the mean age was 12.9 years: differences in developmental stage may therefore have played a role in any differences in results (e.g., more prominent contributions of subcortical structures to social (-cognitive) functioning in our adolescent sample compared to more prominent cortical contributions in their adult sample). A limitation of the present study is its sample size. Although this is comparable to sample sizes of other MRI studies in NF1 [7, 16], the statistical power of certain findings is inherently limited. Whereas a conservative and exhaustive statistical approach was adopted for our volumetric analyses, many correlations between structural volumes and social and executive functioning ratings would not survive further correction for multiple comparisons. It should be noted, however, that the strength of the correlations (plus the bootstrap approach) supports robustness of the results. Considering the pattern of results, particularly with respect to the associations between subcortical volumes and scores on behavior rating scales, it cannot even be ruled out that some type II errors were present.

## Conclusions

Despite its limitations, several conclusions may be drawn from the results of the present study. Most importantly, relatively strong indications were found for the existence of neural correlates of several social and cognitive impairments (as observed by parents) in NF1: in adolescents with NF1, there appears to be either a lack of normal positive associations between volumetric properties of specific brain regions and social/cognitive outcomes or even an inverse relation between the two. These neural substrates and their associations with cognitive-behavioral phenotypes may also be important in other disorders characterized by abnormalities in the Ras/MAPK pathway and synaptic plasticity [30]. Regarding future research directions and potential implications for clinical practice, it should be noted that the etiology of different structural brain abnormalities in NF1 is still very much under investigation. Neurobiological and neuroanatomical characteristics of NF1 should be studied in conjunction, and at different ages, as their interaction may differ depending on developmental stage [2]. Similarly, future studies should combine multiple neuro-imaging techniques in order to provide a more complete picture of the neural substrates underlying cognitive and social functioning in NF1. In the present study, several techniques and means of analyses were already introduced, but we did not include diffusion tensor imaging (DTI), measuring WM integrity, which has also been shown to be affected in NF1 [3]. WM integrity, a measure of structural connectivity, may be the best neural substrate for functional connectivity [32]. Several recent studies showed abnormal resting state functional connectivity in NF1 [33, 34], with further indications that these were associated with cognitive and behavioral impairments (i.e., IQ and self-reported internalizing symptoms) [34].

Thus, in order to obtain insight into relative influence of different aspects of brain structure and function on social and cognitive outcomes in NF1, and subsequently into what might happen following medication or training, it would be advisable to combine neurobiological investigations with a multimodal imaging approach at different stages of development.
